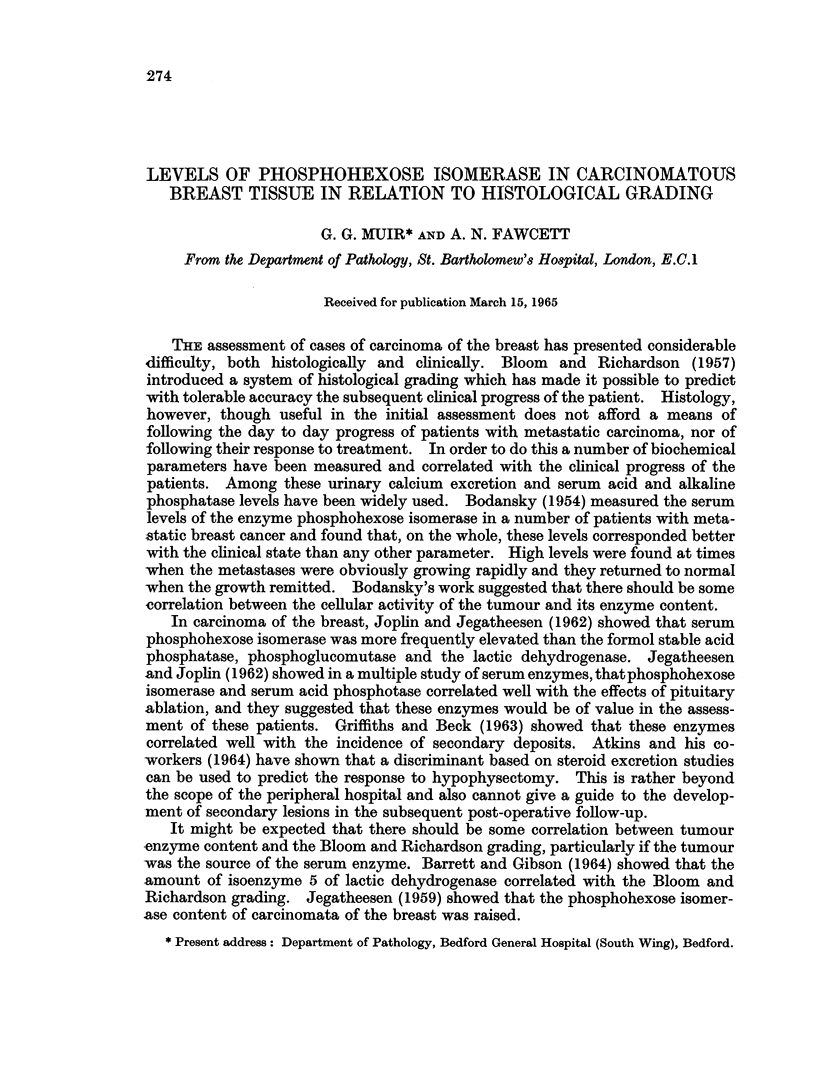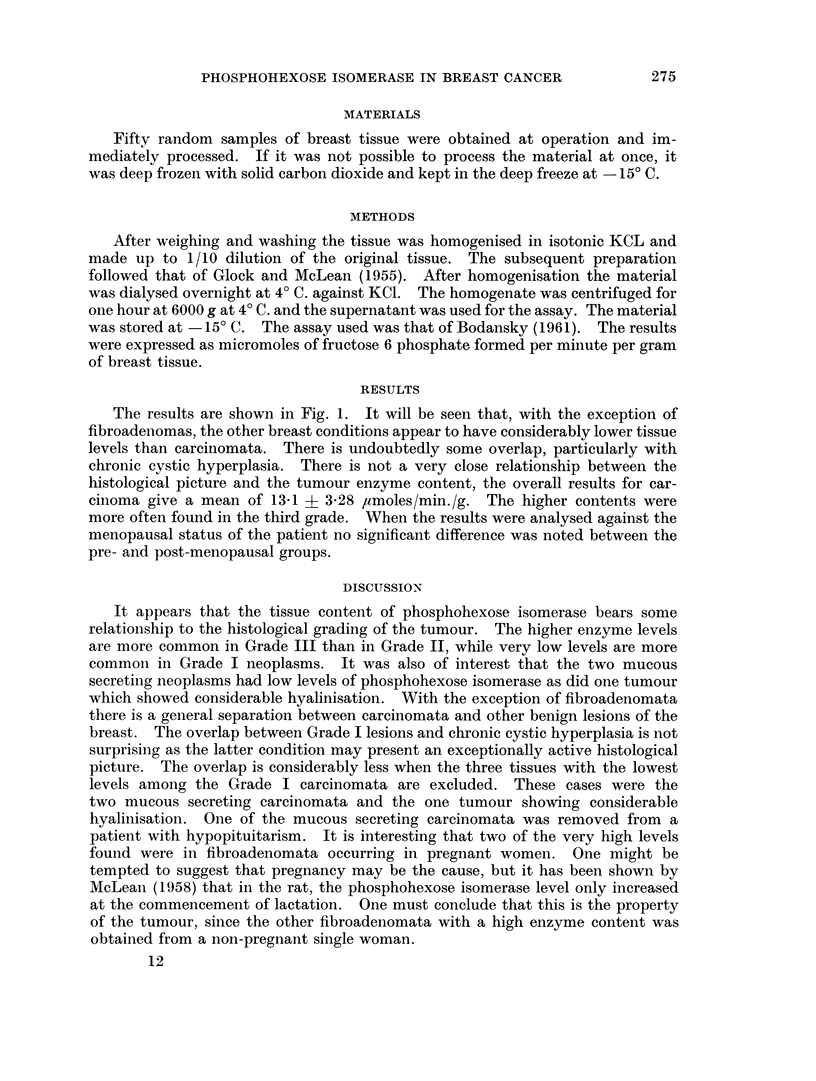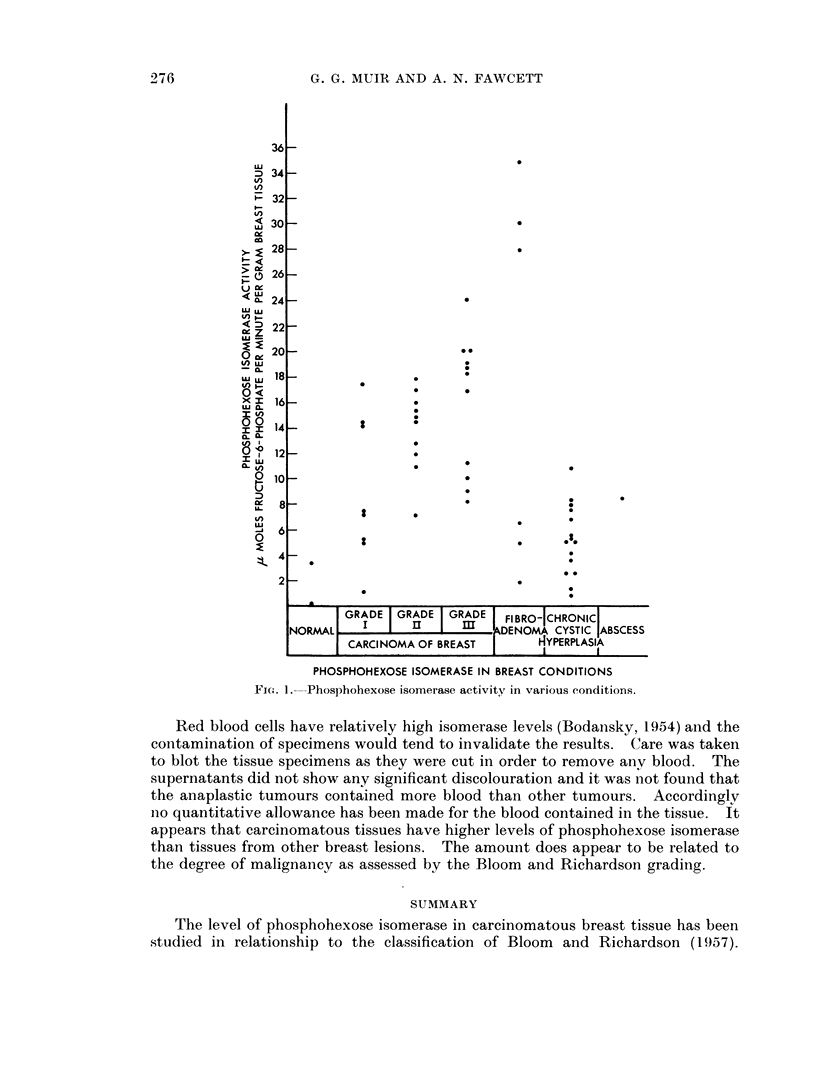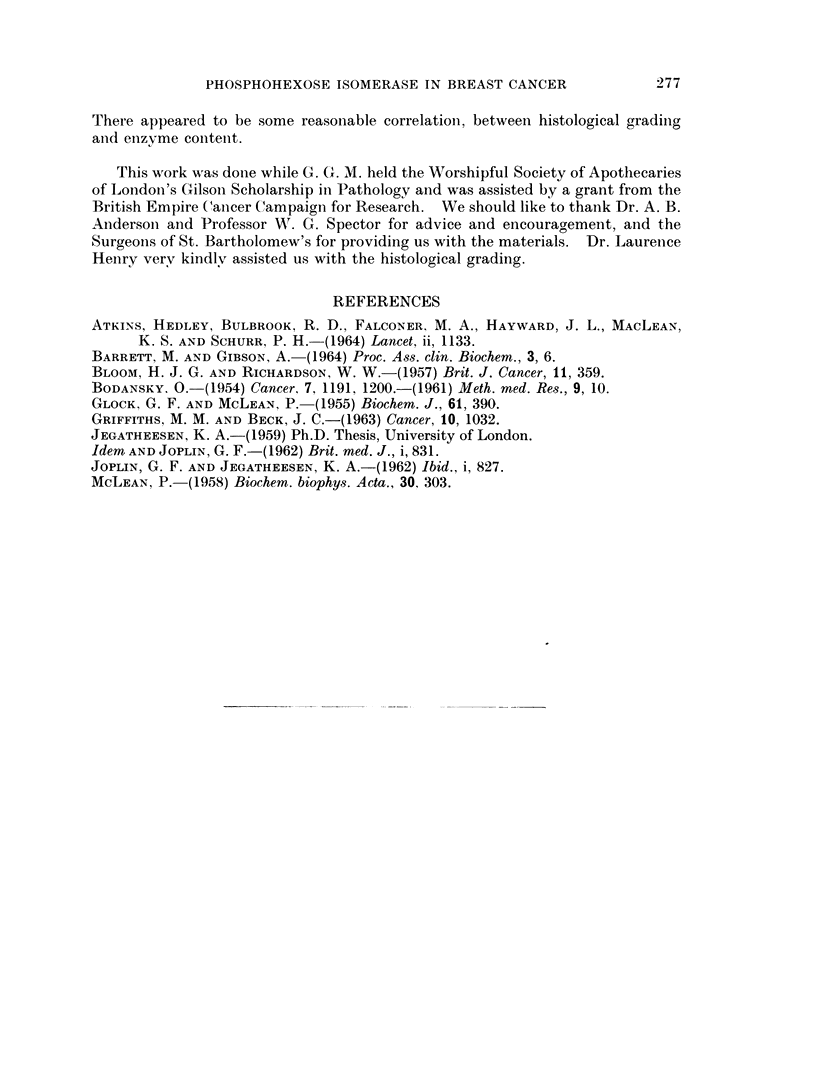# Levels of Phosphohexose Isomerase in Carcinomatous Breast Tissue in Relation to Histological Grading

**DOI:** 10.1038/bjc.1965.31

**Published:** 1965-06

**Authors:** G. G. Muir, A. N. Fawcett


### LEVELS OF PHOSPHOHEXOSE ISOMERASE IN CARCINOMATOUS BREAST TISSUE IN RELATION TO HISTOLOGICAL GRADING

G. G. MUIR* AND A. N. FAWCETT

*From the Department of Pathology, St. Bartholomew's Hospital, London, E.C.*1

Received for publication March 15, 1965

THE assessment of cases of carcinoma of the breast has presented considerable difficulty, both histologically and clinically. Bloom and Richardson (1957) introduced a system of histological grading which has made it possible to predict with tolerable accuracy the subsequent clinical progress of the patient. Histology, however, though useful in the initial assessment does not afford a means of following the day to day progress of patients with metastatic carcinoma, nor of following their response to treatment. In order to do this a number of biochemical parameters have been measured and correlated with the clinical progress of the patients. Among these urinary calcium excretion and serum acid and alkaline phosphatase levels have been widely used. Bodansky (1954) measured the serum levels of the enzyme phosphohexose isomerase in a number of patients with metastatic breast cancer and found that, on the whole, these levels corresponded better with the clinical state than any other parameter. High levels were found at times when the metastases were obviously growing rapidly and they returned to normal when the growth remitted. Bodansky's work suggested that there should be some correlation between the cellular activity of the tumour and its enzyme content.

In carcinoma of the breast, Joplin and Jegatheesen (1962) showed that serum phosphohexose isomerase was more frequently elevated than the formol stable acid phosphatase, phosphoglucomutase and the lactic dehydrogenase. Jegatheesen and Joplin (1962) showed in a multiple study of serum enzymes, that phosphohexose isomerase and serum acid phosphotase correlated well with the effects of pituitary ablation, and they suggested that these enzymes would be of value in the assessment of these patients. Griffiths and Beck (1963) showed that these enzymes correlated well with the incidence of secondary deposits. Atkins and his co-workers (1964) have shown that a discriminant based on steroid excretion studies can be used to predict the response to hypophysectomy. This is rather beyond the scope of the peripheral hospital and also cannot give a guide to the development of secondary lesions in the subsequent post-operative follow-up.

It might be expected that there should be some correlation between tumour enzyme content and the Bloom and Richardson grading, particularly if the tumour was the source of the serum enzyme. Barrett and Gibson (1964) showed that the amount of isoenzyme 5 of lactic dehydrogenase correlated with the Bloom and Richardson grading. Jegatheesen (1959) showed that the phosphohexose isomerase content of carcinomata of the breast was raised.

* Present address : Department of Pathology, Bedford General Hospital (South Wing), Bedford.

### MATERIALS

Fifty random samples of breast tissue were obtained at operation and immediately processed. If it was not possible to process the material at once, it was deep frozen with solid carbon dioxide and kept in the deep freeze at $-15°$ C.

### METHODS

After weighing and washing the tissue was homogenised in isotonic KCL and made up to 1/10 dilution of the original tissue. The subsequent preparation followed that of Glock and McLean (1955). After homogenisation the material was dialysed overnight at 4° C. against KCl. The homogenate was centrifuged for one hour at 6000 *g* at 4° C. and the supernatant was used for the assay. The material was stored at $-15°$ C. The assay used was that of Bodansky (1961). The results were expressed as micromoles of fructose 6 phosphate formed per minute per gram of breast tissue.

### RESULTS

The results are shown in Fig. 1. It will be seen that, with the exception of fibroadenomas, the other breast conditions appear to have considerably lower tissue levels than carcinomata. There is undoubtedly some overlap, particularly with chronic cystic hyperplasia. There is not a very close relationship between the histological picture and the tumour enzyme content, the overall results for carcinoma give a mean of $13{\cdot}1 \pm 3{\cdot}28$ $\mu$moles/min./g. The higher contents were more often found in the third grade. When the results were analysed against the menopausal status of the patient no significant difference was noted between the pre- and post-menopausal groups.

### DISCUSSION

It appears that the tissue content of phosphohexose isomerase bears some relationship to the histological grading of the tumour. The higher enzyme levels are more common in Grade III than in Grade II, while very low levels are more common in Grade I neoplasms. It was also of interest that the two mucous secreting neoplasms had low levels of phosphohexose isomerase as did one tumour which showed considerable hyalinisation. With the exception of fibroadenomata there is a general separation between carcinomata and other benign lesions of the breast. The overlap between Grade I lesions and chronic cystic hyperplasia is not surprising as the latter condition may present an exceptionally active histological picture. The overlap is considerably less when the three tissues with the lowest levels among the Grade I carcinomata are excluded. These cases were the two mucous secreting carcinomata and the one tumour showing considerable hyalinisation. One of the mucous secreting carcinomata was removed from a patient with hypopituitarism. It is interesting that two of the very high levels found were in fibroadenomata occurring in pregnant women. One might be tempted to suggest that pregnancy may be the cause, but it has been shown by McLean (1958) that in the rat, the phosphohexose isomerase level only increased at the commencement of lactation. One must conclude that this is the property of the tumour, since the other fibroadenomata with a high enzyme content was obtained from a non-pregnant single woman.

FIG. 1.—Phosphohexose isomerase activity in various conditions.

Red blood cells have relatively high isomerase levels (Bodansky, 1954) and the contamination of specimens would tend to invalidate the results. Care was taken to blot the tissue specimens as they were cut in order to remove any blood. The supernatants did not show any significant discolouration and it was not found that the anaplastic tumours contained more blood than other tumours. Accordingly no quantitative allowance has been made for the blood contained in the tissue. It appears that carcinomatous tissues have higher levels of phosphohexose isomerase than tissues from other breast lesions. The amount does appear to be related to the degree of malignancy as assessed by the Bloom and Richardson grading.

### SUMMARY

The level of phosphohexose isomerase in carcinomatous breast tissue has been studied in relationship to the classification of Bloom and Richardson (1957).

There appeared to be some reasonable correlation, between histological grading and enzyme content.

This work was done while G. G. M. held the Worshipful Society of Apothecaries of London's Gilson Scholarship in Pathology and was assisted by a grant from the British Empire Cancer Campaign for Research. We should like to thank Dr. A. B. Anderson and Professor W. G. Spector for advice and encouragement, and the Surgeons of St. Bartholomew's for providing us with the materials. Dr. Laurence Henry very kindly assisted us with the histological grading.

### REFERENCES

Atkins, Hedley, Bulbrook, R. D., Falconer, M. A., Hayward, J. L., MacLean, K. S. and Schurr, P. H.—(1964) *Lancet*, ii, 1133.
Barrett, M. and Gibson, A.—(1964) *Proc. Ass. clin. Biochem.*, **3**, 6.
Bloom, H. J. G. and Richardson, W. W.—(1957) *Brit. J. Cancer*, **11**, 359.
Bodansky, O.—(1954) *Cancer*, **7**, 1191, 1200.—(1961) *Meth. med. Res.*, **9**, 10.
Glock, G. F. and McLean, P.—(1955) *Biochem. J.*, **61**, 390.
Griffiths, M. M. and Beck, J. C.—(1963) *Cancer*, **10**, 1032.
Jegatheesen, K. A.—(1959) Ph.D. Thesis, University of London.
*Idem* and Joplin, G. F.—(1962) *Brit. med. J.*, i, 831.
Joplin, G. F. and Jegatheesen, K. A.—(1962) *Ibid.*, i, 827.
McLean, P.—(1958) *Biochem. biophys. Acta.*, **30**, 303.